# Limited value of NT-proBNP as a prognostic marker of all-cause mortality in patients with heart failure with preserved and mid-range ejection fraction in primary care: A report from the swedish heart failure register

**DOI:** 10.1080/02813432.2019.1684029

**Published:** 2019-11-14

**Authors:** Björn Eriksson, Per Wändell, Ulf Dahlström, Per Näsman, Lars H. Lund, Magnus Edner

**Affiliations:** a Department of Neurobiology, Care Sciences and Society (NVS), Division of Family Medicine and Primary Care, Karolinska Institutet, Huddinge, Sweden;; b Department of Cardiology, Linköping University, Linköping, Sweden;; c Department of Medical and Health Sciences, Linköping University, Linköping, Sweden;; d Center for Safety Research, KTH Royal Institute of Technology, Stockholm, Sweden;; e Department of Cardiology, Division of Medicine, Karolinska University Hospital, Karolinska Institutet, Stockholm, Sweden

**Keywords:** Heart failure, EF ≥40%, primary care, NT-proBNP, prognosis

## Abstract

**Aim:** The prognostic value of natriuretic peptides in the management of heart failure (HF) patients with ejection fraction (EF) <40% is well established, but is less known for those with EF ≥40% managed in primary care (PC). Therefore, the aim of this study is to describe the prognostic significance of plasma NT-proBNP in such patients managed in PC.

**Subjects:** We included 924 HF patients (48% women) with EF ≥40% and NT-proBNP registered in the Swedish Heart Failure Registry. Follow-up was 1100 ± 687 days.

**Results:** One-, three- and five-year mortality rates were 8.1%, 23.9% and 44.7% in patients with EF 40–50% (HFmrEF) and 7.3%, 23.6% and 37.2% in patients with EF ≥50% (HFpEF) (*p* = 0.26). Patients with the highest mean values of NT-proBNP had the highest all-cause mortality but wide standard deviations (SDs). In univariate regression analysis, there was an association only between NT-proBNP quartiles and all-cause mortality. In HFmrEF patients, hazard ratio (HR) was 1.96 (95% CI 1.60–2.39) *p* < 0.0001) and in HFpEF patients, HR was 1.72 (95% CI 1.49–1.98) *p* < 0.0001). In a multivariate Cox proportional hazard regression analysis, adjusted for age, NYHA class, atrial fibrillation and GFR class, this association remained regarding NT-proBNP quartiles [HR 1.83 (95% CI 1.38–2.44), *p* < 0.0001] and [HR 1.48 (95% CI 1.16–1.90), *p* = 0.0001], HFmrEF and HFpEF, respectively.

**Conclusion:** NT-proBNP has a prognostic value in patients with HF and EF ≥40% managed in PC. However, its clinical utility is limited due to high SDs and the fact that it is not independent in this population which is characterized by high age and much comorbidity.

**Key points**
- It is uncertain whether NT-proBNP predicts risk in heart failure with preserved ejection fraction (EF > 40%, HFpEF) managed in primary care.
- We show that high NT-proBNP predicts increased all-cause mortality in HFpEF-patients managed in primary care.
- The clinical use is however limited due to large standard deviations, many co-morbidities and high age.
- Many of these co-morbidities contribute to all-cause mortality and management of these patients should also focus on these co-morbidities.

## Introduction

Natriuretic peptides (NPs), commonly BNP or NT-proBNP, are quantitative markers of cardiac dysfunction and are widely recognized as key regulators of blood pressure, water and salt homeostasis [1,2]. They have been widely used in the management of heart failure (HF) during the last 20 years as a ‘rule-out analysis’ in the process of diagnosing HF [3,4]. NPs are mainly produced by cardiovascular, brain and renal tissues in response to wall stretch and other causes, and this is observed both in HF with reduced ejection fraction (HFrEF) and in HF with preserved ejection fraction (HFpEF) [5] In the latest ESC guidelines, patients with an EF in the range of 40–49% are defined as HFmrEF and patients with EF ≥ 50% are defined as HfpEF [6]. It has been shown recently that adherence to guidelines and utilization of NT-proBNP has improved in primary care (PC) over the past years [7].

NPs provide natriuresis, diuresis, vasodilation, antiproliferation, antihypertrophy, antifibrosis and other cardiometabolic protection. The cardiac release of NPs represents an important compensatory mechanism during acute and chronic cardiac overload and during the pathogenesis of HF where their actions counteract the sustained activation of renin-angiotensin-aldosterone and other neurohormonal systems [8]. Elevated circulating plasma NP levels correlate with the severity of HF, and particularly BNP and NT-proBNP have been established as biomarkers for the diagnosis of HF as well as prognostic markers for cardiovascular risk, but they are mainly recommended as a rule-out tool in international guidelines [6,9]. Low values exclude the presence of HF and high values have high positive predictive value for diagnosing HfrEF, but it must be kept in mind that elevated levels of NP are associated with a variety of cardiac (acute pulmonary embolus, acute coronary syndrome, primary pulmonary hypertension) and noncardiac causes (renal failure) and that further diagnostic measurements, preferably echocardiography, often are recommended [10,11].

Several randomized clinical trials (RCTs) have shown that NP-guided treatment has given conflicting results and it is uncertain whether this would lead to a better outcome than simply optimizing treatment according to guidelines [12,13].

The prognostic value of NP levels has been well described and there is a clear connection between high NP levels and poor outcomes of mortality and readmission [14,15]. The results are consistent both for HFrEF-patients and for those with an EF equal to or above 40% when managed in hospital [16,17].

Most studies have been carried out on patients in hospital care (HC). Some studies have been performed on patients in PC but populations have often been mixed [18–20]. Patients with HF are diagnosed and treated in both PC and HC in Sweden even though it has been shown that patients move from one caregiver to another depending on the severity of the disease, and that only 17% are treated exclusively in PC [21]. Furthermore, as stated above, patients with an EF in the range of 40–49% are defined as HFmrEF and patients with EF ≥50% are defined as HFpEF [6]. Because of potential differences concerning these two entities, there is a need for more studies.

Therefore, the aim of this study is to assess the prognostic significance of plasma NT-proBNP in patients with HFmrEF and HFpEF managed in PC.

## Methods

### Study protocol

The Swedish Heart Failure Registry (SwedeHF) has previously been described in detail [22]. Unselected patients with HF are prospectively registered in PC and in HC at an out-patient visit, or on discharge from hospital. The inclusion criterion is clinician-judged HF. This is based on typical symptoms (e.g. breathlessness, ankle swelling and fatigue) and findings (e.g. elevated jugular venous pressure, pulmonary crackles and peripheral edema). However, diagnosis based only on this is uncertain and should be confirmed with echocardiography. We have therefore chosen, in our study, to include only patients who had undergone echo, especially since we wanted to find patients with EF ≥40% that can only be measured with echo. Approximately 80 variables are recorded and entered into a web-based database managed by Uppsala Clinical Research Center (Uppsala, Sweden). The protocol, registration form and annual reports are available at http://www.rikssvikt.se. Individual patient consent is not required but patients are informed of entry into the national registry and allowed to opt out. The registry and this study were approved by a multisite ethic committee (Dnr 2013/444-32) and conform to the Declaration of Helsinki.

In a study in 2018, we used data from SwedeHF on patients with HFpEF in PC- and HC-based out-patient clinics to describe characteristics and outcomes in a population with HF and EF ≥ 40%. In that study, we used data from the SwedeHF recorded between 1 September 2001 and 15 May 2014 after the database had been merged with the Swedish population register and the Swedish patient register of hospitalization. The two latter registries are governed by the Swedish Board of Health and Welfare. Sweden had 1156 PC units and 78 hospitals in 2014. Of these, 116 PC units and 67 hospitals participate in the register. In total, 59,075 unique patients, 6579 from PC and 52,496 from HC, were eligible for the study. We included only patients registered at an out-patient visit either in PC or in HC. We excluded patients without information about echocardiography (1041 = 15.8% in PC and 5938 = 11.3% in HC) and in the next step patients with an EF <40% as well as hospitalized HF patients with an EF ≥40% who entered the registry before hospital discharge. Thus, 1802 patients registered at a PC-based out-patient clinic visit and 7852 patients registered at a HC-based out-patient clinic, all with an EF of more than or equal to 40%, remained in the study. The complete population has been previously described in detail [23].

**Figure 1.**
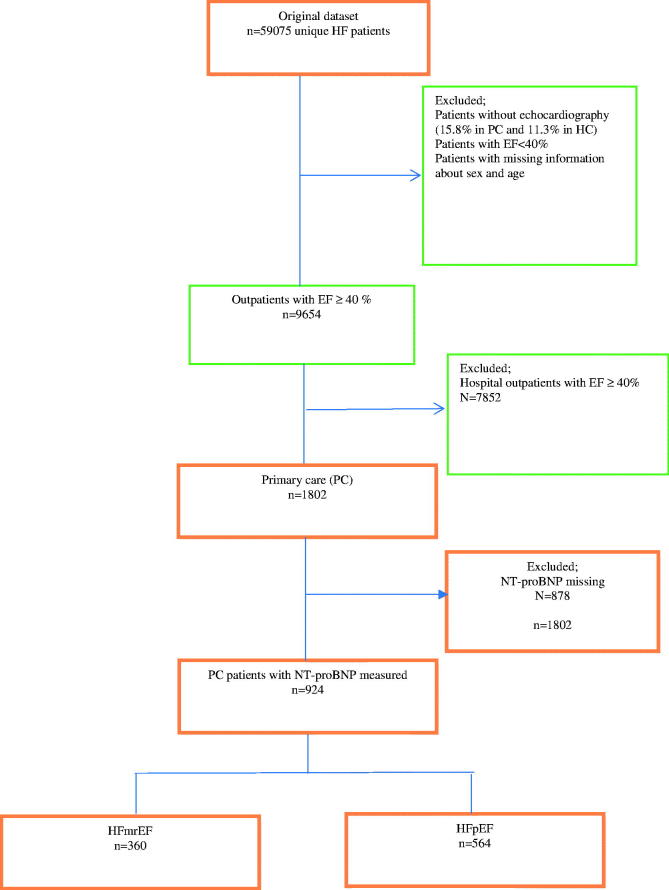
Schematic patient selection.

For the present study, we included only patients registered in PC who had a measurement of NT-proBNP registered (924 of 1802, 51%). The study flow is shown in detail in Figure 1.

In Sweden, echocardiography is the recommended method for defining left ventricular EF. Four categories are used in the SwedeHF: EF <30, 30–39, 40–49 and ≥50%

Renal function was assessed as estimated glomerular filtration rate (e-GFR) and calculated according to the formula of MDRD and divided into four different classes, <30, 30–59, 60–89 and ≥90 µmol/l/min.

NT-proBNP values (ng/L) are presented as mean with standard deviation (SD) and median with quartiles.

### Statistical analyses

Descriptive statistics were presented as numbers (n) and percentages (%) or means with SD and compared with Chi-Square or Student’s unpaired *t*-tests as appropriate.

Crude outcomes in HFmrEF and HFpEF separately were assessed with Kaplan–Meier analysis.

Cox regression analyses were performed for NT-proBNP and the different variables considering elapsing time, calculating hazard ratios (HRs) for mortality, in order to reassure that NT-proBNP was an independent risk factor. We performed a univariate regression analysis for mortality in a first step to find variables which all had a *p*-value of 0.1 or below before entering these in a Cox regression multivariate analyses.

All statistical analysis were performed using SAS 9.4. The level of significance was 5% and levels of significance were set at a *p*-value *≤0.05, **<0.01 and ***<0.0001. All *p*-values and confidence intervals were two-sided.

## Results

### Characteristics at registration in SwedeHF

Between 1 September 2001 and 15 May 2014, there were 59,075 unique patients registered in SwedeHF. After exclusions, 924 HF patients with an EF equal to or above 40%, and with NT-proBNP measured, remained in the study (Figure 1). All patients were managed in PC by general practitioners (GP’s) and all patients were registered at an out-patient visit.

Mean follow-up time was 1100 ± 687 days. The patients were divided into two groups, 360 with EF 40–49% (HFmrEF) and 564 patients with EF ≥50% (HFpEF) (Table 1).

**Table 1.** Characteristics at registration of patients with heart failure and EF ≥40% in SwedeHF, all managed in primary care (*n* = 924).

	HFmrEF (EF 40–49%) *n* = 360	HFpEF (EF > 50%) *n* = 564	*p* Value
Male, n (%)	221 (61)	260 (46)	***
Female, n (%)	139 (39)	304 (54)	***
Age, mean (SD)	76.3 (9.6)	78.2 (8.5)	**
Dead, n (%)	113 (31.4)	173 (30.7)	
Smoking, n (%)	28 (7.8)	26 (4.6)	
Weight, kg (SD)	82.5 (17.2)	80.9 (18.4)	
IHD total, n (%)	146 (40.6)	202 (35.8)	
IHD verified with angiography, n (%)	55 (17.5)	75 (14.3)	
Hypertension, n (%)	228 (64)	390 (70)	
Atrial fibrillation, n (%)	195 (54.8)	286 (50.8)	
Diabetes, n (%)	73 (20.3)	109 (19.3)	
COPD, n (%)	81 (22.6)	141 (25.4)	
Valvular disease, n (%)	74 (20.6)	136 (24.1)	
CABG and/or PCI, n (%)	38 (11.1)	37 (6.9)	**
CRT, n (%)	1 (0.3)	0	
ECG Sinus rhythm, n (%)	169 (47.6)	312 (56.6)	**
Pulse frequency, mean bpm (SD)	71.9 (14.8)	72.1 (13.2)	
Systolic blood pressure, mean mm Hg (SD)	134.0 (20.5)	135.9 (21.1)	
Diastolic blood pressure, mean mm Hg (SD)	75.4 (11.4)	75.5 (11.1)	
Hemoglobin, mean g/l (SD)	135.2 (15.2)	132.6 (15.3)	
Creatinine, mean micromol/l (SD)	100.4 (31.5)	96.9 (35.1)	
eGFR < 30, n (%)	15 (4.2)	27 (4.8)	
eGFR 30–59, n (%)	157 (43.6)	246 (43.6)	
eGFR 60–89, n (%)	159 (44.2)	225 (39.9)	
eGFR > 90, n (%)	29 (8.1)	66 (11.7)	
eGFR, mean ml/min (SD)	61.9 (19.1)	63.3 (21.1)	
NT-proBNP, ng/L (SD)	2508.7 (3420.3)	2140.3 (2950.0)	
Chest X-ray normal, n (%)	93 (40.1)	202 (52.6)	**
NYHA 1, n (%)	35 (14.3)	39 (13.2)	
NYHA 2, n (%)	135 (55.3)	168 (56.8)	
NYHA 3, n (%)	69 (28.3)	83 (28.0)	
NYHA 4, n (%)	5 (2.1)	6 (2.0)	
ACEi, n (%)	216 (60.0)	283 (50.2)	**
ARB, n (%)	110 (30.6)	173 (30.7)	
BB, n (%)	285 (79.2)	410 (73.0)	*
Diuretics, n (%)	103 (28.6)	162 (28.8)	
MRA, n (%)	67 (18.7)	115 (20.4)	
Digitalis, n (%)	42 (11.7)	72 (12.8)	
Statins, n (%)	183 (51.1)	240 (42.6)	*

ACEi: ACE-inhibitors; ARB: Angiotensin receptor blockers ARB; BB: Beta blockers; CABG: Coronary artery bypass grafting; COPD: Chronic obstructive pulmonary disease; CRT: Cardiac resynchronization therapy; ICD: Implantable cardioverter defibrillator; IHD: Ischemic heart disease; IHD: Ischemic heart disease; NYHA: New York Heart Association; MRA: Mineral corticoid receptor antagonist; NYHA: New York Heart Association; PCI: Percutaneous coronary intervention.

*p Value <0.05.

**p Value <0.01.

***p Value <0.0001.

Patients with HFmrEF were slightly younger and more likely of male gender. They had more often been through an intervention against ischemic heart disease [coronary artery bypass (CABG) or percutaneous coronary intervention (PCI)] and less frequently had a sinus rhythm on ECG or a normal chest X-ray. They were also more often treated with ACE-inhibitors, beta-blockers and statins. Detailed figures are presented in Table 1.

### The prognostic value of plasma-NT-proBNP

**Figure 2.**
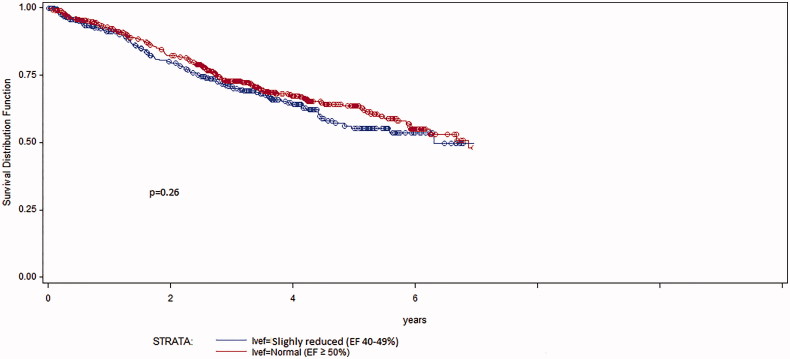
Kaplan–Meier survival curves illustrating all-cause mortality in HFmrEF patients (blue) and HFpEF patients (red) during follow-up 1100 ± 687 days (*p* = 0.26).

**Figure 3.**
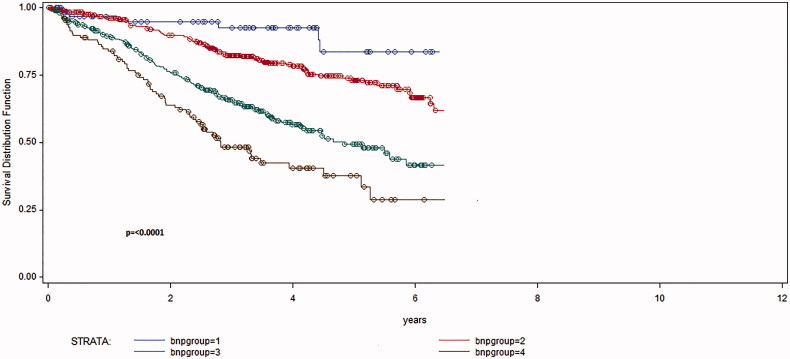
Kaplan–Meier survival curves illustrating all-cause mortality in the four NT-proBNP quartiles during follow-up 1100 ± 687 days (p= <0.0001).

One-, three- and five-year mortality rates were 8.1%, 23.9% and 44.7% for patients with HFmrEF and 7.3%, 23.6% and 37.2% for patients with HFpEF (*p* = 0.26) (Figure 2). Patients with the highest mean values of NT-proBNP had the highest all-cause mortality but wide SDs (Table 2). In univariate regression analysis, there was an association only between NT-proBNP quartiles and all-cause mortality (Figure 3).

**Table 2.** Mean values of NT-proBNP ng/L ± SD for patients that died after 1, 3 and 5 years.

	1 year	3 years	5 years
EF >40	5076.8 (5524.4)	3935.3 (4402.6)	3721.9 (4317.3)
EF 40–49	5133.2 (5473.4)	4169.3 (4006.0)	4052.6 (4244.6)
EF ≥50	5035.9 (5630.3)	3785.1 (4647.7)	3493.2 (4365.5)

#### HFmrEF (EF 40–49%)

NT-proBNP quartiles ranges were: 0–780, 781–1492, 1493–2919 and >2920. As for the entire study population, patients with HFmrEF patients who belonged to the highest NT-proBNP quartiles had the highest all-cause mortality. In a univariate regression analysis there was a highly statistically significant association between NT-proBNP quartiles and all-cause mortality. HR 1.96 (95% CI 1.60–2.39) *p*-value < 0.0001. In a multivariate Cox proportional hazard regression analysis adjusted for variables which in the univariate regression analysis had a *p*-value of 0.1 or lower (age, NYHA class, atrial fibrillation and GFR class) NT-proBNP remained highly associated with all-cause mortality with HR 1.83 (95% CI 1.38–2.44) *p*-value <0.0001 (Table 3).

**Table 3.** Cox proportional hazard regression analysis for association between NT-proBNP quartiles and all-cause mortality in HFmrEF patients.

Outcome mortality	HR	95% CI	*p* Value
Univariate	1.96	1.60–2.39	<0.0001
Adjusted only age	1.74	1.41–2.15	<0.0001
Adjusted only NYHA class	1.77	1.36–2.31	<0.0001
Adjusted only atrial fibrillation	2.08	1.68–2.58	<0.0001
Adjusted only eGFR	1.82	1.48–2.23	<0.0001
Adjusted age and NYHA class	1.68	1.28–2.22	0.0002
Adjusted age, NYHA class and atrial fibrillation	1.84	1.38–2.44	<0.0001
Adjusted age, NYHA class, atrial fibrillation and eGFR	1.83	1.38–2.44	<0.0001
Adjusted age, NYHA class, atrial fibrillation, eGFR, MRA, statins and COPD	1.84	1.38–2.46	<0.0001

One patient with NT-proBNP below 100 ng/L died during the follow-up time.

#### HFpEF (EF ≥ 50%)

NT-proBNP quartiles ranges were 0–524, 525–1254, 1255–2528 and >2529. As for the entire study population, patients with HFpEF who belonged to the highest NT-proBNP quartile had the highest mortality. In a univariate regression analysis, there was a highly statistically significant association between NT-proBNP quartile level and all-cause mortality. HR 1.72 (95% CI 1.49–1.98), *p*-value < 0.0001. In a multivariate Cox proportional hazard regression analysis adjusted for variables that in a univariate regression analysis had *p*-value 0.1 or less (age, NYHA class, atrial fibrillation and GFR class) NT-proBNP remained highly associated with all-cause mortality with HR 1.48 (CI 1.16–1.90), *p*-value =0.0001 (Table 4).

**Table 4.** Cox proportional hazard regression analysis for association between NT-proBNP quartiles and all-cause mortality in HFpEF patients.

Outcome mortality	HR	95% CI	*p* Value
Univariate	1.72	1.49–1.98	<0.0001
Adjusted only age	1.59	1.38–1.84	<0.0001
Adjusted only NYHA class	1.49	1.19–1.87	0.0006
Adjusted only atrial fibrillation	1.82	1.56–2.11	<0.0001
Adjusted only eGFR	1.65	1.43–1.91	<0.0001
Adjusted age and NYHA class	1.38	1.10–1.74	0.0059
Adjusted age, NYHA class and atrial fibrillation	1.50	1.18–1.92	0.0010
Adjusted age, NYHA class, atrial fibrillation and eGFR	1.48	1.16–1.90	0.0017
Adjusted age, NYHA class, atrial fibrillation, eGFR, MRA, statins and COPD	1.70	1.30–2.23	<0.0001

Three patients with NT-proBNP below 100 ng/L died during follow-up.

### Variables associated with increased NT-proBNP

#### HFmrEF (EF 40–49%)

We performed a univariate regression analysis to find variables that were associated with a higher level of NT-proBNP. Variables with a *p*-value ≤ 0.1 for an elevated level of NT-proBNP were age, NYHA class, hemoglobin level, systolic blood pressure, diastolic blood pressure and body weight. Thus, these variables were significant in a univariate regression analyses. In the following multivariate Cox proportional hazard regression analysis age and low hemoglobin level remained significantly associated with higher NT-proBNP level.

#### HFpEF (EF ≥ 50%)

We performed a univariate regression analysis to find variables that were associated with a higher level of NT-proBNP. Variables with a *p*-value ≤ 0.1 for an elevated level of NT-proBNP were age, NYHA class, hemoglobin level, diastolic blood pressure, body weight, valvular disease, atrial fibrillation, diabetes and kidney function. Thus, these variables were significant in a univariate analyses. In the following multivariate Cox proportional hazard regression analysis valvular disease and low body weight remained significantly associated with higher NT-proBNP level.

### Comorbidities and characteristics affecting all-cause mortality

There was a high frequency of comorbidities in the two EF groups. Only 3% of HFmrEF patients and 2% of HFpEF patients had no registered comorbidity. Previous or current hypertension was the most common comorbidity. This occurred in 64% of the patients in the HfmrEF group and 70% of those belonging to the HfpEF group (Table 1). More than 50% in both EF groups had atrial fibrillation. These diseases were often combined with one or more other diseases. The most common combination of comorbidities was previous or current hypertension and atrial fibrillation (8% in both EF groups). The combination of chronic obstructive pulmonary disease (COPD) and hypertension was more than twice as common in patients with HFpEF compared with those with HFmrEF.

In HFmrEF patients age, low body weight, low diastolic blood pressure, low hemoglobin level, low creatinine clearance class and NYHA class were associated with higher all-cause mortality in a univariate regression analysis taking into account variables with *p*-value ≤0.1. However, in a multivariate Cox proportional hazard regression analysis only NYHA class remained highly significantly associated with all-cause mortality [HR 2.09 (CI 1.37–3.18), *p* = 0.0006].

In HFpEF patients age, low body weight, low diastolic blood pressure, low hemoglobin level, creatinine clearance class, COPD, valvular disease and NYHA class were associated with higher all-cause mortality in a univariate regression analysis taking into account variables with *p*-value ≤0.1. However, in a multivariate Cox proportional hazard regression analysis only age [HR 1.07 (95% CI 1.02–1.12)], low body weight [HR 0.98 (95% CI 0.96–1.00)], COPD [HR 2.13 (95% CI 1.21–3.74)] and NYHA class [HR 1.67 (95% CI 1.08–2.59)] remained statistically significantly associated with all-cause mortality.

## Discussion

We have studied 924 HF patients with midrange (HFmrEF) and preserved (HFpEF) EF managed in PC who had plasma NT-proBNP registered. Our intention was to evaluate the prognostic significance of plasma NT-proBNP in these two groups. The main finding is that there is a strong association between elevated NT-proBNP expressed as quartiles and all-cause mortality but that the clinical utility of measuring NT-proBNP in this population is limited due to large SDs. Moreover, different comorbidities are important but COPD was common and affected all-cause mortality in patients with HFpEF. Other factors of importance in this study were high age and low body weight. Our results also indicate that the NYHA class gives enough information in most cases.

### Characteristics at registration in SwedeHF

Patients in the HFmrEF group were younger and more often of male gender than those in the HFpEF group. They were more often treated with ACE-inhibitors (ACEi), beta blockers and statins, even though there was no statistically significant difference concerning comorbidity between the two groups. Despite this, they had more often been through an intervention such as CABG or PCI, indicating that they suffered from more ischemic heart disease. Perhaps the difference concerning medication reflects that patients in the HFmrEF group sometimes were judged as having HfrEF, in which case this treatment would be appropriate. However, there was no difference in all-cause mortality despite the difference in medical treatment. Patients with HfpEF, on the other hand, had more comorbidities in total, above all hypertension, COPD and valvular disease. Furthermore, they were older, reflecting the complexity of managing these patients in a PC setting. In other studies, patients with HFpEF have consistently been found to be older, more often female, more predominantly hypertensive, and to have a higher prevalence of atrial fibrillation and with a lower prevalence of coronary artery disease than those with HFrEF [24]. Notably, non-cardiovascular co-morbidities also appear to be highly prevalent in HFpEF, consistent with an elderly population, and include renal impairment, chronic lung diseases, anemia, cancer, liver disease, peptic ulcer disease and hypothyroidism [24–27]. Further, patients with HFpEF more often die of non-cardiac causes than patients with HFrEF. The high frequency of comorbidities in our study may partly explain the difficulty in using NT-proBNP as a prognostic tool, since many of these comorbidities can independently affect the NT-proBNP value [25,28].

### The prognostic value of plasma-NT-proBNP

It is previously known that there is a clear association between high NP levels and poor outcome of mortality and readmission, and that the results are consistent for both HFrEF and HfpEF [16,17]. However, most of these studies have been carried out on patients treated in hospital, whereas there are only a few studies on PC patients [18–20].

In our study, there was a clear, highly statistically significant, association between NT-proBNP, measured in quartiles, and all-cause mortality. Even after an adjusted multivariate Cox proportional hazard regression analysis, NT-proBNP quartiles remained strongly associated with all-cause mortality. This is in accordance with what has previously been shown for patients in HC, but not yet for patients with HFmrEF and HFpEF in PC [14,15].

Thus, in our study NT-proBNP appeared particularly unspecific, which seriously limited the clinical utility. Even though there was a strong association between NT-proBNP, as measured in quartiles, and all-cause mortality, the SDs were high. In everyday clinical practice, this means that it will be difficult to use NT-proBNP as a prognostic marker for the separate patient case. A high NT-proBNP value in a specific patient case will probably mean a bad prognosis. However, a low value does not exclude a bad prognosis. In a PC setting with elderly patients and multimorbidity, the practical use of a single NT-proBNP analysis as a prognostic marker of all-cause mortality is therefore of doubtful clinical importance. We know, as pointed out in the introduction, that NT-proBNP is well established as a rule-out analysis in the diagnostic process of HF, but the question has also been whether we can use the analysis as a prognostic tool, helping us to concentrate on risk categories. Our results in this study indicate that this is not the case. NYHA classification, which is simple and cheap but poorly used, seems to be as good an alternative.

### Variables associated with increased NT-proBNP

NT-proBNP is released from cardiomyocytes as a response to increased wall stretch and is an important compensatory mechanism during acute and chronic cardiac overload. Elevated circulating plasma NT-proBNP levels correlate with the severity of HF [8]. However, NT-proBNP levels are also elevated by other conditions such as pulmonary embolus, acute coronary syndrome, primary pulmonary hypertension, age and renal dysfunction [10,11]. PC patients with HFmrEF and HFpEF have a high level of comorbidities, and in our study only 3% of HFmrEF patients, and 2% of HFpEF patients, had no registered comorbidity. The most common comorbidities were previous or current hypertension and atrial fibrillation. This must be kept in mind when using the unspecific NT-proBNP as a prognostic tool.

In this study, we found that age and low hemoglobin level remained significantly associated with a higher NT-proBNP after multivariate analysis in the HFmrEF group, and valvular disease and low body weight in the HFpEF group. In conclusion, many factors influence levels of NT-proBNP, and the clinical implications of the elevated levels are difficult to interpret. Several confounding factors, such as aging, obesity, anemia, sepsis, hypertension, MI, cardiac hypertrophy, pulmonary hypertension, atrial fibrillation, diabetes mellitus, renal dysfunction, liver cirrhosis, severe burn injuries and cancer chemotherapy have been described as limiting the accuracy of NPs [1].

### Comorbidities affecting all-cause mortality

As expected, we found a clear association between NYHA classification and mortality in both groups, higher NYHA class having higher all-cause mortality. High NYHA classification is a reason for further investigation and could, judging by our findings, be useful as a prognostic tool. However, it is unfortunately not always registered [23].

Another important finding in this study is the high association between COPD and all-cause mortality in the HFpEF group. As COPD is often managed in PC, and furthermore easily underdiagnosed, due to its slow progress and sometimes vague symptoms, it appears important to actively exclude it. Since there is no evidence-based medical therapy for HFpEF today, managing COPD and anemia as well as maintaining normal body weight in this group is of great importance, not least due to the elderly population and a complex comorbidity panorama. In a recently published study, Zafrir et al. [29] showed that the prevalence of atrial fibrillation increases with increasing EF, and that its association with worse cardiovascular outcomes remained significant in patients with HFmrEF and HFpEF, but not in those with HFrEF. However, in the present study, we could not observe any correlation between atrial fibrillation and mortality.

Furthermore, in both HF groups optimizing blood pressure and treating anemia is crucial, especially since both actions in many cases are relatively easy to perform. Great attention must also be paid to the kidney function in these elderly patients, often with polypharmacy.

The causes of death, according to the national registry, were in our study: cardiovascular in 57%, malignant tumors in 14% and respiratory diseases in 11%, further stressing the need to monitor and treat all problems affecting the patient. Grimsmo et al. have also recently discussed the complexity of treating patients with multimorbidity in PC [30].

The high association between NYHA class and all-cause mortality in both groups, compared with NT-proBNP, underlines the usability of NYHA classification as a base for prognostic information.

## Strengths and limitations

The SwedeHF is one of the largest HF registries in the world. The size of the registry, both in the number of patients and in the amount of variables together with nationwide use, yields generalizability and unique possibilities to study large cohorts of HF patients. The opportunity to connect this register to other Swedish national registries, such as those of death and hospitalization via the Swedish individual personal number system, adds to the potential advantages.

However, participating in SwedeHF is not mandatory in Sweden. Therefore, there is a risk that PC units reporting to the registry often are more interested in HF and more dedicated to managing HF patients and following the guidelines. Of Sweden’s 1156 PC units only 116 (10%) participated in SwedeHF, which underlines this explanation. The corresponding rate for hospitals was 67 out of 78 hospitals (86%). This may lead to a selection of PC units not being representative of Swedish PC in general. Possibly the PC cohort in the present study might show better results than a study of PC units, in general, would do.

Further, the registry does not provide information on all possible comorbidities. There is information about previous or current hypertension, COPD, ischemic heart disease, atrial fibrillation, diabetes, kidney function, anemia and valvular disease but not about cancer and other serious diseases that may influence outcome and prognosis.

In this study, we have no information on whether the diagnosis of HFpEF has been thoroughly established according to the ESC classification in the different GP offices, and therefore we have classified them merely based on their EF. The diagnosis requires evidence of either relevant structural heart disease or diastolic dysfunction, none of which we can obtain from the database. This is a clear limitation, as well as the fact that we do not know exactly when in the clinical course the NT-proBNP values was examined. However, we know that samples most often, according to local routines, are taken in conjunction with the visit.

## Conclusions

NT-proBNP is a risk factor associated with increased all-cause mortality in patients with EF 40–49% (HFmrEF) and ≥50% (HFpEF) but its clinical usefulness is limited due to its unspecific character. It could not be claimed as an independent risk factor. Only 57% of the patients in this population have a cardiovascular cause of death, and both EF groups are characterized by large heterogeneity, with many comorbidities and high age. Management of patients should also focus on these conditions.

## Data Availability

BE, PW and ME had full access to all of the data in the study and take responsibility for the integrity of the data and the accuracy of the data analysis.
